# Baikalsky State Nature Biosphere Reserve and its buffer zone: floristic data

**DOI:** 10.3897/BDJ.10.e76946

**Published:** 2022-01-06

**Authors:** Natalia Sergeevna Gamova

**Affiliations:** 1 Herbarium (MW), Faculty of Biology, M. V. Lomonosov Moscow State University, Moscow, Russia Herbarium (MW), Faculty of Biology, M. V. Lomonosov Moscow State University Moscow Russia; 2 Baikalsky State Nature Biosphere Reserve, Tankhoy, Russia Baikalsky State Nature Biosphere Reserve Tankhoy Russia

**Keywords:** vascular plants, occurrence, Khamar-Daban Range, Republic of Buryatia, Russia

## Abstract

**Background:**

Baikalsky State Nature Biosphere Reserve is situated in the central part of the Khamar-Daban Range (Southern Baikal, Siberia), in three administrative districts of Republic of Buryatia (i.e. Kabansky District, Dzhidinsky District and Selenginsky District), Russia. In general, this territory has been relatively well studied by botanists, but until now there was no detailed information about the flora of the Reserve with precise geographic localities. Moreover, some records in the Baikalsky Reserve's flora were published without references to documenting herbarium specimens.

**New information:**

The dataset contains 39,238 unique occurrences of 875 taxa (854 species, 14 subspecies, five varieties and two species aggregates) from the Baikalsky Reserve and its buffer zone. All the data were acquired during the field studies by the author in 2009–2021, when 152 taxa (17.3% of all the taxa included into the dataset) were first recorded by the author from the study area. Herbarium vouchers are preserved in the Moscow University Herbarium (MW). This dataset is the first attempt at creating a database of vascular plants of the Baikalsky Reserve and its buffer zone, based on modern research. These data will provide the background for the updated check-list of the Baikalsky Reserve's flora.

## Introduction

Baikalsky Reserve was established in 1969 to protect local plant communities of the Khamar-Daban Range within all the altitudinal zones. The Baikalsky Reserve's flora includes several relict plant species (Epova 1956, Chepinoga et al. 2017b). To protect the unique natural complexes of this area, Baikalsky Reserve was assigned the status of Biosphere Reserve in 1986. Therefore, a special buffer zone surrounding its borders was created (buffer zone is obligatory for all the Biosphere Reserves in Russia). The Baikalsky Reserve is one of the key natural protected areas in the system of nature conservation in southern Siberia. The Reserve is also a part of the Lake Baikal World Heritage Site since 1996 (Unesco World Heritage Center (2021)).

First detailed botanical studies here began in the middle of the 20^th^ century. Scientists from Irkutsk described the vegetation of the Khamar-Daban Range and collected herbarium specimens (herbarium vouchers are preserved now in the Herbaria IRK and IRKU). N.A. Epova studied the vegetation and relict species and made the first map of floristic division of this territory (Epova 1956, Epova 1957, Epova 1960, Epova 1961). M.M. Ivanova continued this work and also explored the alpine flora of the Khamar-Daban Range (Ivanova 1967). The first check-list of vascular plants of the Baikalsky Reserve was published in 1978 (Vasilchenko et al. 1978) and contained 777 taxa. Floristic investigations continued and several additions to the flora were published later (Volotovsky and Ermolenko 1985, Ivanova 1991, Krasnopevtseva et al. 2008a). The second check-list was published in 2008 (Krasnopevtseva et al. 2008b) and contained 886 taxa; new additions followed soon (Abramova et al. 2009). The third check-list, including 991 taxa, was published in 2011 (Abramova and Volkova 2011). Several additions to the flora of the Baikalsky Reserve and its buffer zone were published since 2011 (Gamova and Dudov 2012, Krasnopevtseva and Krasnopevtseva 2012, Gamova and Krasnopevtseva 2013, Verkhozina et al. 2013, Ivanova et al. 2016, Sutkin et al. 2016, Tupitsyna and Chepinoga 2016, Gamova and Dudov 2018a, Gamova and Dudov 2018b, Gamova et al. 2018a, Gamova et al. 2018b, Chepinoga et al. 2019, Gamova et al. 2019, Sutkin and Krasnopevtseva 2020). In 2009–2021, 152 new taxa were first recorded by the author and added to the flora of the Baikalsky Reserve and its buffer zone. For other 18 new taxa, firstly found in recent years by other botanists, the author has recorded new localities.

Open-access data with precise geographic references are essential for modern research as it allows a computational approach to analysing spatial patterns of species distributions. This is the first comprehensive dataset focusing on the flora of the Baikalsky Reserve covering its whole territory and all the taxonomic groups of vascular plants. Several datasets, including some data from the territory of the Baikalsky Reserve amongst other areas, were published in recent years (Chepinoga et al. 2017a, Brianskaia et al. 2021, Seregin 2021). However, these datasets focused on various aspects, namely on the distribution of alpine endemic plants of northern Asia, on digitising grid maps from the “Flora of Central Siberia” (1979) or on digitising herbarium collections of Moscow State University, neither of them aimed at thoroughly studying the Baikalsky Reserve's flora. Thus, the aim of the present work is to narrow this gap in the knowledge of the vascular plant species distributions in the Baikalsky Reserve.

## General description

### Purpose

The main purpose of the dataset (Gamova 2021) is to give access to the modern floristic data from the Baikalsky Reserve, which is situated in a hard-to-reach area in the central part of the Khamar-Daban Range and to study the plant diversity of this territory. Systematic data collection enabled us to reveal the local species richness.

### Additional information

The dataset contains 39,238 occurrences from 3,156 sampling events. A total of 90.39% of the dataset (35,467 occurrences) is formed by species lists from 1,873 relevés (59.35% of all sampling events). The number of species per one relevé varies from 8 to 87, with 19 species on average.

The flora of the Baikalsky Reserve has been actively studied since the Reserve was established, but only recently has it become possible to create a database containing exact geographic localities. Furthermore, both current species distributions and full check-list of the flora of the Baikalsky Reserve may require further verification.

The dataset aims at biodiversity monitoring and inventory in the framework of scientific research in the Baikalsky Reserve. This dataset does not cover a full list of vascular plants of the Baikalsky Reserve. It contains the data collected only by the author during her own field research. All the herbarium vouchers preserved at the Moscow University Herbarium (MW) have been made available online via GBIF (Seregin 2021). All findings of the taxa reported by the author for the first time for the Baikalsky Reserve and its buffer zone are documented with herbarium vouchers. This herbarium collection contains both common and rare species from the territory (Fig. 1).

At present, the dataset contains 875 taxa at species and infraspecific levels. This is approximately 280 species less than in our preliminary unpublished check-list of the Baikalsky Reserve's flora which includes all records from all the published sources. Revisiting the herbarium collections in Irkutsk, Ulan-Ude and Tomsk revealed that ca. 150 species out of these 280 have never been documented by herbarium vouchers and their publications cannot be confirmed. All the herbarium specimens from our territory kept at the Novosibirsk Herbarium were mentioned in the check-list of the flora of Baikal's shores (Popov and Busik 1966), lacking the aforementioned 150 species, as well. Therefore, published reports without herbarium vouchers should be interpreted with caution. To date, only some records were rejected after our re-examination of herbarium specimens when original identifications were erroneous; for example, *Alliumsplendens*, *Carexselengensis*, *Hypericumascyron* etc. were re-identified and those species were excluded (Gamova et al. 2019) from our preliminary check-list. The presence of other species mentioned in earlier publications, but not confirmed by herbarium specimens, will be further verified via fieldwork.

As the majority of herbarium specimens collected by other researchers and not included in this dataset date back to the middle of the 20^th^ century, they lack the geographic coordinates and the textual description of the locality does not allow georeferencing with the precision of 1–2 km. Sometimes, the description is even more vague and the uncertainty reaches 5–10 km. Approximately 30 species, recently recorded by other researchers and confirmed by herbarium specimens with geographic coordinates and, therefore, suitable for this type of dataset, were not included. Thus, the dataset with the original data collected by the author contains the majority of vascular plant species authentically known from Baikalsky Reserve and its buffer zone.

At the moment, a new check-list of the vascular plant flora of the Baikalsky Reserve, based on modern data and herbarium specimens, is being compiled. The data from the other herbaria will be further included into the updated check-list and georeferenced with the maximum available precision.

## Sampling methods

### Study extent

The Baikalsky Reserve with its buffer zone is situated mostly in the mountains. The lowest point of this territory is situated on the Baikal shore (457 m a.s.l.) and the highest point is Mt. Sokhor (2,316.7 m a.s.l.) in the middle part of the Baikalsky Reserve. The Temnik River forms the southern border of the Baikalsky Reserve (1,100–830 m a.s.l. from west to east). Altogether the territory of the Baikalsky Reserve and its buffer zone covers ca. 2,000 km^2^ (167,871 ha and 34,788 ha, respectively).

The Khamar-Daban Range is one of the mountain chains framing Lake Baikal in its rift zone; its origin dates back to the Baikal Orogeny (650–550 million years). Despite the ancient age, alpine landforms are widespread here due to mountain glaciation. The territory of the Baikalsky Reserve belongs to the area with local distribution of permafrost; some permafrost spots are located in the highlands and on the southern side (Gerasimov 1965).

The climate of the northern side of the Khamar-Daban Range in its central part is relatively mild for southern Siberia. The mean annual temperatures vary from -0.3°C at Tankhoy meteostation (460 m a.s.l.) to -3.4°C at the Khamar-Daban meteostation (1,420 m a.s.l.). The mean July temperatures are +14.7°C and +12.7°C; the mean January temperatures are -17°C and -17.9°C, correspondingly. The mean annual amount of precipitation is 900–1,450 mm. Winter snow cover reaches at least 90–100 cm with an average depth of 150 cm and the maximum depth of 190–200 cm (Ladeishchikov et al. 1977). Both the annual amount of precipitation and the winter snow cover in the central part of the northern side of the Khamar-Daban Range are the maximal in the Baikal Region. The climate of the southern side is drier (400–600 mm annual precipitation) and more continental. The mean annual temperature here is about -5°C; the mean July temperature is +12°C and the mean January temperature is -22°C. The average duration of the vegetation period varies from 120–150 days on the Baikal shore to 100 days on the main watershed of the Khamar-Daban Range (Kartushin 1969).

According to "Zones and altitudinal zonality types of vegetation of Russia and adjacent territories", the study area within the central part of the Khamar-Daban Range belongs to the Khamar-Daban variant (*Abiessibirica* and *Pinussibirica* forests, *Pinuspumila* elfin woods) of Tuva–Southern Transbaical group (nival–goltsy–tundra–taiga) of the Boreal (Taiga) type of altitudinal zonality (Ogureeva et al. 1999). The territory of the Baikalsky Reserve includes all the diversity of local altitudinal zonality (Epova 1957, Epova 1961, Tjulina 1976). The northern side is covered by coniferous forests (Siberian taiga with *Pinussibirica* and *Abiessibirica*) in its main part. Subalpine meadows and elfin woods of dwarf Siberian pine (*Pinuspumila*) are situated above the timber line. Vegetation of the main watershed of the Khamar-Daban Range consists mostly of mountain tundra and alpine meadows. The altitudinal zonality of the southern side is presented by three main vegetation belts. Subalpine meadows are less presented here and *Pinuspumila* elfin woods are well-developed. The upper forest belt here is presented by *Pinussibirica*, while the most part of the southern side is covered by larch taiga (*Larixsibirica*) and its lower part – by pine taiga (*Pinussylvestris*). Some local stations of steep rocky slopes in the valley of the Temnik River are very dry and steppe plant communities are situated here. In addition, there are forests (including secondary ones) with bogs and meadows which are widespread along the Baikal shores. Intrazonal vegetation of river valleys is represented by mixed polydominant forests with *Populussuaveolens* mostly on the northern side of the Khamar-Daban Range.

The flora of the Khamar-Daban Range belongs to the Altay-Sayan Province of the Circumboreal Region of the Boreal (Holarctic) floristic kingdom, following Takhtajan (1986). The flora of the Baikalsky Reserve and its buffer zone by its origin consists of two main parts: native species and alien ones. Although the alien section of the flora reaches ca. 120–130 species (12% of total flora), it is concentrated in the narrow belt (0.5–4 km) of the buffer zone adjacent to the northern border of the Baikalsky Reserve near the Baikal shore. Several settlements (the major one is Tankhoy, a village with ca. 950 inhabitants, where the headquarters of the Baikalsky Reserve are situated), roads (highway Irkutsk – Ulan-Ude and the Trans-Siberian Railway) and communications (power transmission line) are situated also in the northern part of the buffer zone near Baikal. The western, southern and eastern parts of the buffer zone of the Baikalsky Reserve are situated in the mountains and are not anthropogenically disturbed. According to the “Atlas of Russia's intact forest landscapes” (Aksenov et al. 2003), the core zone of the Baikalsky Reserve is formally classified as intact. The world map of intact forest landscapes (Potapov et al. 2021) indicating forest losses in 2000–2013, 2013–2016 and 2016–2020, shows that no significant changes in the forest cover occurred in the recent years. Therefore, alien species are not widespread here. Only less than 10 taxa of alien species (*Chaenorhinumminus*, *Conyzacanadensis*, *Epilobiumadenocaulon*, *E.pseudorubescens*, *Impatiensglandulifera*, *Puccinelliahauptiana*, *Senecioviscosus*, *S.vulgaris*) are relatively common (or have become common in recent years) in the buffer zone of Baikalsky Reserve, while the majority of them have rare or single occurrences (Gamova et al. 2018b). The flora of the Baikalsky Reserve includes several endemic or sub-endemic species. One of the most famous endemic species is *Swertiabaicalensis* (Gentianaceae). It is known only from the western and central parts of the Khamar-Daban Range and the Baikalsky Reserve protects the main part of its distribution area. One of the new taxa – *Eranthistanhoensis* (Ranunculaceae) – was described in 2020 (Erst et al. 2020). This species was named after Tankhoy Village. It is also an endemic of southern Baikal Region (Fig. 2).

### Sampling description

All the data were obtained during fieldwork in summer seasons 2009–2021 (except 2020 due to the COVID-related lockdown), mostly between 10 June and 20-25 August, in some years also including the beginning of September. This time interval covers the major part of the vegetation development in this region. Ephemerous plant species are absent from the Baikalsky Reserve and ephemeroid plants begin to flower in May and continue in June. All of them remain noticeable until mid-July or August; thus, this group of species is adequately represented in the dataset. In general, the field works were conducted during 2–2.5 months per year, except 2011 when field data were collected only for three weeks.

The dataset contains information from different sources:

1. Relevés made in various altitude belts and plant communities. The locations for the relevés were chosen carefully within the contours of homogeneous plant communities. The area of the relevés was 400 m^2^ for forest communities and 100 m^2^ for herbaceous ones. If the area of the plant community was less than those mentioned above, the community was described within its natural limits. The original data also contain additional information on the projective cover of species etc., but only the species list was included in the dataset.
2. Plants collected as herbarium specimens. In all the cases, when species identification in the field was impossible and/or new taxa for the Baikalsky Reserve were recorded, specimens were preserved.
3. Field notes about plant occurrences. Additional localities for particular species, mostly rare and endangered ones, or remarkable localities for common species (for example, single trees above the timber line etc.).

The research was conducted systematically, the field routes being planned to access the maximal coverage of different parts of the Baikalsky Reserve and its buffer zone. The major part of the Baikalsky Reserve's territory is located in the mountains. The only highway (Irkutsk – Ulan-Ude) is situated north of Baikalsky Reserve. As the rest of the territory is inaccessible for motor transport, it was explored mostly on foot; pack horses were also used on the lower part of the southern side and half of the length of the Temnik River is accessible via airboat. Within the borders of the Baikalsky Reserve, four mountain passes (1,400–2,220 m a.s.l.) across the Khamar-Daban Range are predominantly used. The majority of paths follow the river valleys. To evenly study the territory, the routes were planned along the paths and radially around field camp sites. The total length of field routes comprised ca. 320 km in 2021; in the other years, it varied from 180 km to 420 km.

All the data reflect the spontaneous distribution of plants, both native and alien species. Cultivated plants from Tankhoy Village and other minor populated places situated in the buffer zone of the Baikalsky Reserve are not included into the dataset.

### Quality control

All the localities were georeferenced in the field via portable GPS-navigators Garmin (Etrex H in 2009–2017 and GPSmap 64 st since 2018), so the accuracy of the coordinates was limited by the technical characteristics of the devices.

Most of the plant identifications were made by the author, but there are several cases in specific taxonomic groups, in which identification was made by taxonomic experts and local florists. These are the following taxa: Boraginaceae, Chenopodiaceae, *Artemisia*, *Calamagrostis*, *Carex*, *Epilobium*, *Hieracium*, *Oxytropis*, *Persicaria*, *Pilosella*, *Poa*, *Polygonum*, *Potamogeton* and *Salix*. As a result, 37,532 occurrences (95.65% of the dataset) were identified to the species or infraspecific levels and 274 occurrences (0.7%) were referred to two species aggregates, i.e. *Festucaovina* and *Taraxacumofficinale*.

To reveal the local vascular plant diversity to its full extent, the occurences, identified to the genus level, were also included into the dataset (i.e. 1432 occurrences or 3.65% of the dataset, for 53 genera). *Alchemilla*, *Euphorbia*, *Hedysarum*, *Lepidium*, *Sinapis*, *Sparganium* and *Valeriana* were identified to the genus level only. Other 46 genera have occurrences both at species and genus levels. These are mostly *Allium*, *Betula*, *Calamagrostis*, *Carex*, *Salix* and some others. In most cases, though, the choice for unidentified species could be considerably narrowed to a certain selection according to the check-lists of the flora of the Republic of Buryatia and Irkutsk Region (Anenkhonov et al. 2001, Chepinoga et al. 2008); such variants are mentioned below in alphabetical order of genera. Cases, where too many options are possible due to the species richness within the genus (as in genera *Carex* and *Salix*) or where new alien species can be found (*Alchemilla*) or for some other reasons, are noted as "unknown".

*Achillea*: *Ptarmica* group. *Aconitum*: blue-flowered species (*A.ambiguum*, *A.baicalense*, *A.glandulosum*; perhaps some others). *Agrostis*: *A.clavata*, *A.tenuis*; perhaps *A.trinii*. *Alchemilla*: unknown; this group needs a special revision in Baikal Region due to recent findings of several alien species. *Allium*: non-flowering plants of *A.splendens* and *A.strictum*; *A.amphibolum*, *A.malyschevii*, perhaps some others, depending on habitat and altitude. *Alopecurus*: non-flowering plants, most possibly *A.arundinaceus*. *Aquilegia*: non-flowering plants of *A.glandulosa* or *A.sibirica*. *Avena*: most possibly *A.fatua* or *A.sativa*, perhaps some other alien species. *Betula*: *B.pubescens* or *B.platyphylla* in cases, where key features for identification were not available. *Brassica*: unknown; except *B.campestris*; possibly, *B.juncea*. *Calamagrostis*: *C.arundinacea*, *C.lapponica*, *C.neglecta*, *C.pavlovii*, *C.tenuis*, depending on habitat and altitude. *Carex*: unknown. *Cerastium*: mostly alpine species; *C.flavescens*, *C.pusillum*, perhaps some others. *Chenopodium*: unknown, other than *C.album*. *Deschampsia*: unknown, other than *D.cespitosa*. *Elymus*: *E.caninus*, *E.confusus*, *E.mutabilis*, *E.transbaicalensis*. *Eriophorum*: alpine species; most possibly *E.brachyantherum*, *E.gracile*, *E.humile*, perhaps some others. *Euphorbia*: either *E.borealis* or *E.discolor*. *Euphrasia*: most possibly *E.pectinata* or *E.stricta*. *Gastrolychnis*: unknown. *Glyceria*: *G.lithuanica*, *G.plicata*, *G.spiculosa*, *G.triflora*. *Gymnocarpium*: either *G.dryopteris* or *G.jessoense*. *Hedysarum*: *H.alpinum*, *H.inundatum*, *H.neglectum*, perhaps some others. *Hieracium*: unknown. *Hierochloe*: possibly *H.odorata* or *H.glabra*. *Huperzia*: alpine plants different from *H.selago* s.str.; perhaps, *H.appressa*. *Lappula*: *L.redowskii* or *L.squarrosa*. *Lepidium*: unknown; possibly, *L.densiflorum* or *L.virginianum*. *Lonicera*: either L.caeruleasubsp.pallasii or L.caeruleasubsp.altaica; all plants from the southern macroslope, due to uncertainty of features in many cases. *Luzula*: alpine species, other than *L.kamtschadalorum*. *Lycopodium*: either *L.clavatum* or *L.lagopus* in subalpine zone. *Myosotis*: unknown, other than *M.cespitosa*, *M.imitata* or *M.scorpioides*. *Pilosella*: unknown. *Plantago*: *P.depressa*, *P.major* or *P.media*. *Poa*: non-flowering plants; most possibly species within the list of species mentioned in the dataset. *Potamogeton*: unknown; most possibly species within the list of species mentioned in the dataset. *Pulsatilla*: other than *P.orientali-sibirica* or *P.turczaninovii*; perhaps, *P.bungeana*. *Pyrola*: non-flowering plants, most possibly *P.incarnata* or *P.minor*. *Ranunculus*: most possibly *R.polyanthemos* or *R.propinquus*; perhaps some other species close to these two. *Rhizomatopteris*: either *R.montana* or *R.sudetica*. *Salix*: unknown shrub species, most possibly others than mentioned in the dataset. *Saussurea*: *S.latifolia* or *S.parviflora* in habitats where both species can possibly occur. *Sinapis*: *S.alba* or *S.arvensis*. *Sparganium*: unknown. *Spiraea*: non-flowering plants of either *S.flexuosa* or *S.media*. *Stellaria*: alpine species, unknown. *Taraxacum*: local alpine species, possibly *T.ceratophorum*, *T.mongolicum*. *Trisetum*: *T.agrostideum* or *T.altaicum* for alpine habitats; for others most possibly *T.sibiricum*. *Urtica*: most possibly, *U.dioica* or *U.galeopsifolia*. *Valeriana*: *V.alternifolia* (if separated from *V.officinalis*) or *V.transjenisensis*. *Vicia*, *Viola*: species within the list of mentioned in the dataset. *Woodsia*: unknown; most possibly others than *W.ilvensis*; perhaps *W.glabella*, *W.calcarea* or some other species.

## Geographic coverage

### Description

**Figure 3.**
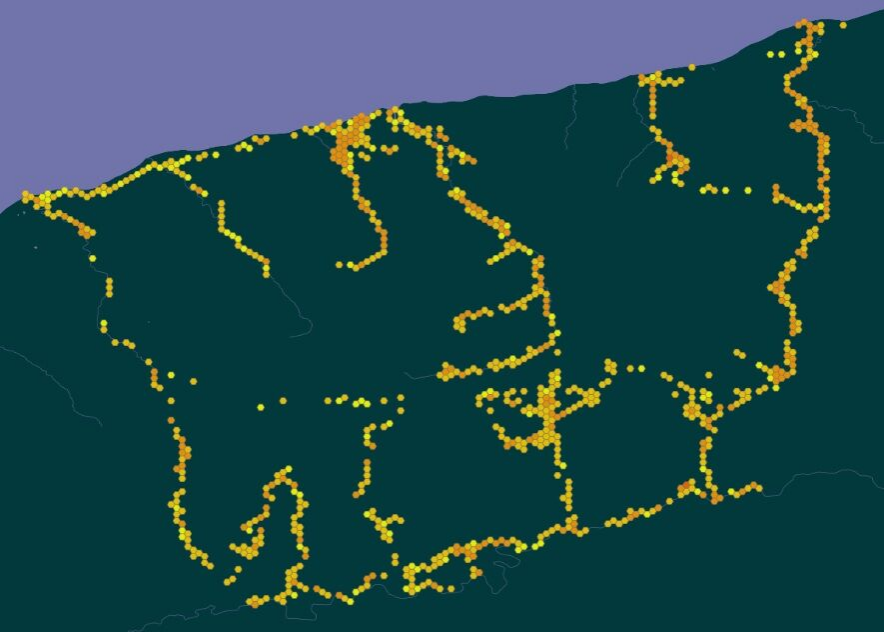
Geographic coverage of records within the dataset (Baikalsky State Nature Biosphere Reserve and its buffer zone: floristic data available via GBIF on https://doi.org/10.15468/g4sdvs).

In general, all the territory of the Baikalsky Reserve and its buffer zone is covered by our field routes. The records are concentrated mainly along the paths and river valleys or some localities of particular botanical interest (Fig. 3).

### Coordinates

51.1 and 51.7 Latitude; 104.8 and 105.6 Longitude.

## Taxonomic coverage

### Description

The dataset includes all the Tracheophyta found in the Baikalsky Reserve and its buffer zone. The majority of occurrences belong to Magnoliopsida (25,580 or 65.19%); Liliopsida has 6,979 occurrences (17.79%), Pinopsida – 3,678 occurrences (9.38%), Polypodiopsida – 2,430 occurrences (6.19%), Lycopodiopsida – 564 occurrences (1.44%), and Gnetopsida (with its only member *Ephedramonosperma*) – seven occurrences (0.01%).

The taxa included into the dataset belong to 410 genera of 89 plant families. Top-10 families with the largest numbers of taxa at the species or infraspecific level and the largest number of occurrences are mentioned in Tables 1, 2. The taxa from the top-10 plant families shown in Table 1 include 502 taxa (57.37% of the taxonomic diversity included into the dataset). The occurrences of plants from the top-10 plant families shown in Table 2 include 23,272 occurrences (59.31% of the dataset).

Although Pinaceae does not belong to the top-10 plant families with the largest number of taxa, it has the largest number of occurrences (3,534) in the dataset.

### Taxa included

**Table:** 

Rank	Scientific Name	
phylum	Tracheophyta	

## Traits coverage

### Data coverage of traits

PLEASE FILL IN TRAIT INFORMATION HERE

## Temporal coverage

**Data range:** 2009-6-25 – 2021-8-30.

### Notes

**Figure 4.**
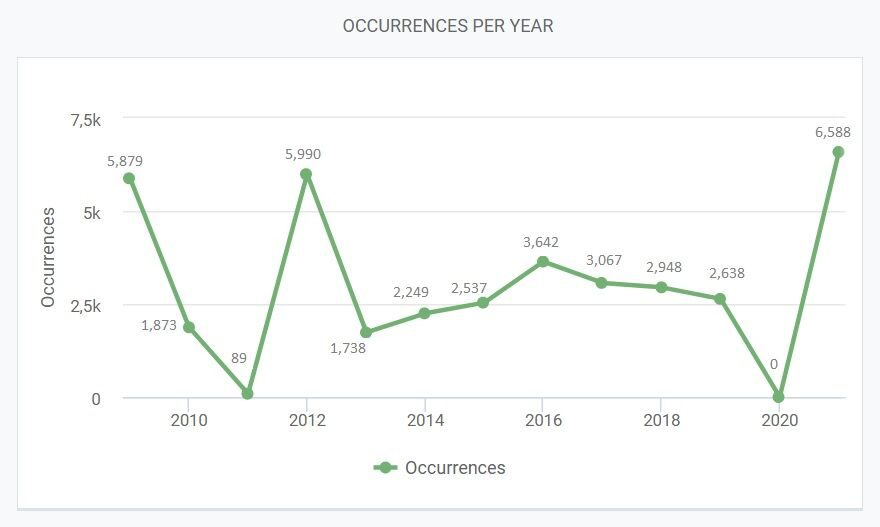
Occurrences per year.

Distribution of records by year is shown in Fig. 4.

## Usage licence

### Usage licence

Creative Commons Public Domain Waiver (CC-Zero)

### IP rights notes

This work is licensed under a Creative Commons Attribution (CC-BY) 4.0 License.

## Data resources

### Data package title

Baikalsky State Nature Biosphere Reserve and its buffer zone: floristic data

### Resource link

https://doi.org/10.15468/g4sdvs

### Alternative identifiers

10.15468/g4sdvs,11512fe2-50cf-48e1-b681-3bb1f347eb86, https://depo.msu.ru/ipt/resource?r=baikal

### Number of data sets

1

### Data set 1.

#### Data set name

Baikalsky State Nature Biosphere Reserve and its buffer zone: floristic data

#### Data format

Darwin Core

#### Number of columns

41

#### Description

The dataset contains records on vascular plants from Baikalsky State Nature Biosphere Reserve and its buffer zone. The Reserve is situated in three admimistrative districts of Republic of Buryatia (i.e. Kabansky District, Dzhidinsky District and Selenginsky District), Russia. Field data were obtained in 2009–2021. Herbarium vouchers are preserved in Moscow University Herbarium (MW).

**Data set 1.** 

Column label	Column description
occurrenceID	An identifier for the occurrence. A variable constructed from a combination of two identifiers (datasetID and catalogNumber). For example, "urn:lsid:biocol.org:col:15550:12:00001".
dcterms:type	The nature or genre of the resource. A constant ("Dataset").
dcterms:modified	The most recent date-time on which the resource was changed. A constant ("2021-11-25").
dcterms:language	A language of the resource. A constant ("en" = English)
dcterms:licence	A legal document giving official permission to do something with the resource. A constant. ("http://creativecommons.org/licenses/by/4.0/legalcode")
dcterms:rightsHolder	A person or organisation owning or managing rights over the resource. A constant ("Moscow State University").
dcterms:accessRights	Information about who can access the resource or an indication of its security status. A constant ("Use under CC BY 4.0").
institutionID	An identifier for the institution having custody of the object(s) or information referred to in the record. A constant ("http://grbio.org/institution/moscow-stateuniversity" for the Moscow State University).
collectionID	An identifier for the collection or dataset from which the record was derived. A constant ("urn:lsid:biocol.org:col:15550" for the Moscow University Herbarium).
datasetID	An identifier for the set of data. May be a global unique identifier or an identifier specific to a collection or institution. A constant ("urn:lsid:biocol.org:col:15550:12").
institutionCode	The name (or acronym) in use by the institution having custody of the object(s) or information referred to in the record. A constant ("Moscow State University").
datasetName	The name identifying the dataset from which the record was derived. A constant ("Baikalsky State Nature Biosphere Reserve and its buffer zone: floristic data").
ownerInstitutionCode	The name (or acronym) in use by the institution having ownership of the object(s) or information referred to in the record. A constant ("Moscow State University").
basisOfRecord	The specific nature of the data record - a subtype of the dcterms:type. A constant ("HumanObservation").
informationWithheld	Additional information that exists, but that has not been shared in the given record. A variable. For example, "Associated ecological data and frequency estimate", "Voucher reference".
catalogNumber	An identifier (preferably unique) for the record within the dataset or collection. A variable. For example, "00001".
recordedBy	A list (concatenated and separated) of names of people, groups or organisations responsible for recording the original occurrence. A constant ("Natalia S. Gamova").
occurrenceStatus	A statement about the presence or absence of a taxon at a location. A constant ("present").
eventID	An identifier for the set of information associated with an event. A variable. For example, "2009-001".
eventDate	The date when the event was recorded. A variable. For example, "2009-06-25".
higherGeography	A list (concatenated and separated) of geographic names less specific than the information captured in the locality term. A constant ("Asia | Russian Federation | Republic of Buryatia").
continent	The name of the continent in which the location occurs. A constant ("Asia").
country	The name of the country or major administrative unit in which the location occurs. A constant ("Russian Federation").
countryCode	The standard code for the country in which the location occurs. A constant ("RU").
stateProvince	The name of the next smaller administrative region than country (state, province, canton, department, region etc.) in which the location occurs. A constant ("Republic of Buryatia").
minimumElevationInMetres	The lower limit of the range of elevation (altitude, above sea level), in m a.s.l. A variable. For example, "648".
decimalLatitude	The geographic latitude (in decimal degrees, using the spatial reference system given in geodeticDatum) of the geographic centre of a Location. A variable. For example, "51.5670".
decimalLongitude	The geographic longitude (in decimal degrees, using the spatial reference system given in geodeticDatum) of the geographic centre of a Location. A variable. For example, "105.3956".
geodeticDatum	The ellipsoid, geodetic datum or spatial reference system (SRS) upon which the geographic coordinates given in decimalLatitude and decimalLongitude are based. A constant ("WGS84").
coordinateUncertaintyInMetres	The horizontal distance (in metres) from the given decimalLatitude and decimalLongitude describing the smallest circle containing the whole of the location. A constant ("10").
coordinatePrecision	A decimal representation of the precision of the coordinates given in the decimalLatitude and decimalLongitude. A constant ("0.0001").
georeferencedBy	A list (concatenated and separated) of names of people, groups or organisations who determined the georeference (spatial representation) of the location. A constant ("Natalia S. Gamova").
georeferencedDate	Same as eventDate; georeferenced with GPS during observation. A variable. For example, "2009-06-25".
georeferenceSources	A list (concatenated and separated) of maps, gazetteers or other resources used to georeference the Location, described specifically enough to allow anyone in the future to use the same resources. A constant ("field GPS data").
identifiedBy	A list (concatenated and separated) of names of people, groups or organisations who assigned the Taxon to the subject. A constant ("Natalia S. Gamova").
scientificName	The full scientific name, with authorship and date information, if known. A variable. For example, "*Abiessibirica* Ledeb.".
kingdom	The full scientific name of the kingdom in which the taxon is classified. A constant ("Plantae").
phylum	The full scientific name of the phylum or division in which the taxon is classified. A constant ("Tracheophyta").
taxonRank	The taxonomic rank of the most specific name in the scientificName. A variable (five options: "species", "subspecies", "genus", "variety", "species aggregate").
nomenclaturalCode	The nomenclatural code (or codes in the case of an ambiregnal name) under which the scientificName is constructed. A constant ("International Code of Nomenclature for algae, fungi and plants").
taxonomicStatus	The status of the use of the scientificName as a label for a taxon. A constant ("accepted").

## Additional information

Gamova N S (2021). Baikalsky State Nature Biosphere Reserve and its buffer zone: floristic data. Version 1.6. Lomonosov Moscow State University. Occurrence dataset https://doi.org/10.15468/g4sdvs accessed via GBIF.org on 2021-11-25.

## Figures and Tables

**Figure 1a.**
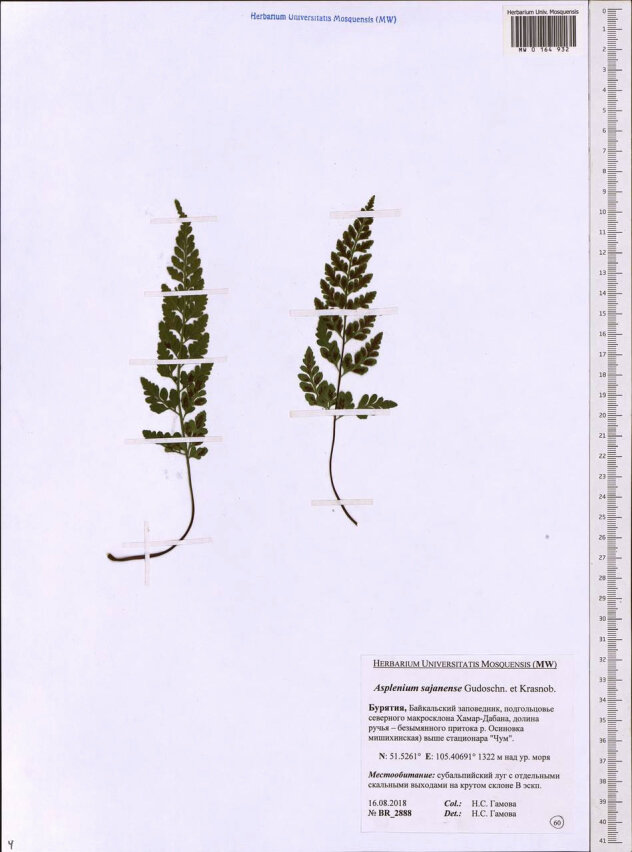
*Aspleniumsajanense*. Herbarium voucher from MW.

**Figure 1b.**
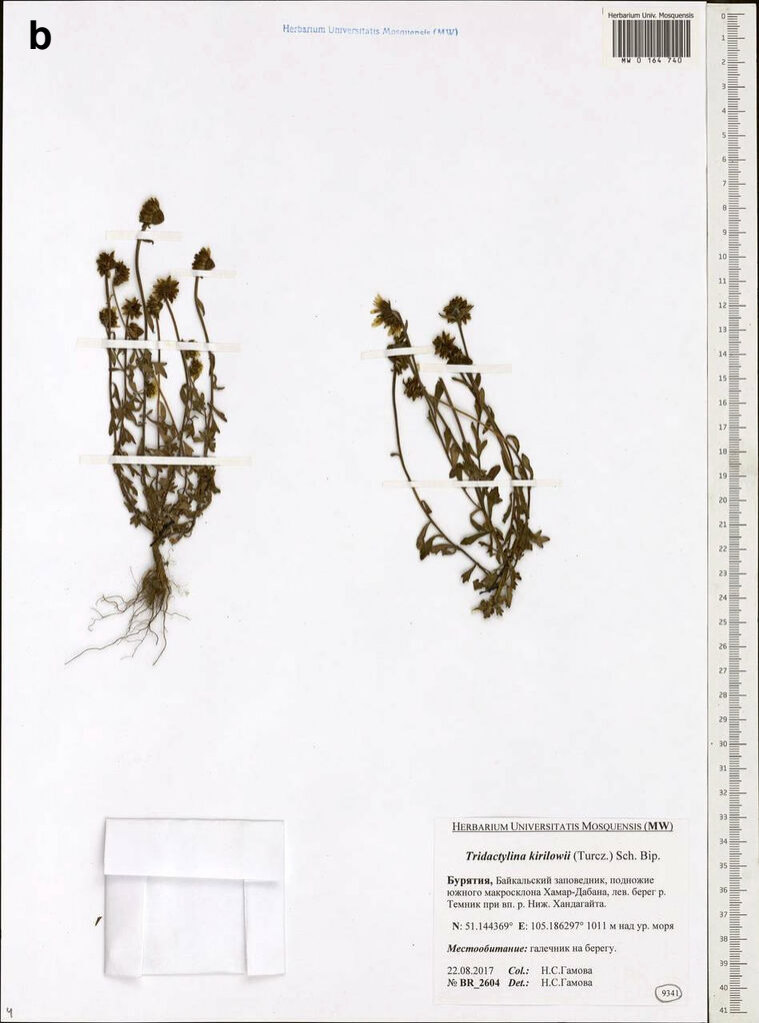
*Tridactylinakirilowii*. Herbarium voucher from MW.

**Figure 2a.**
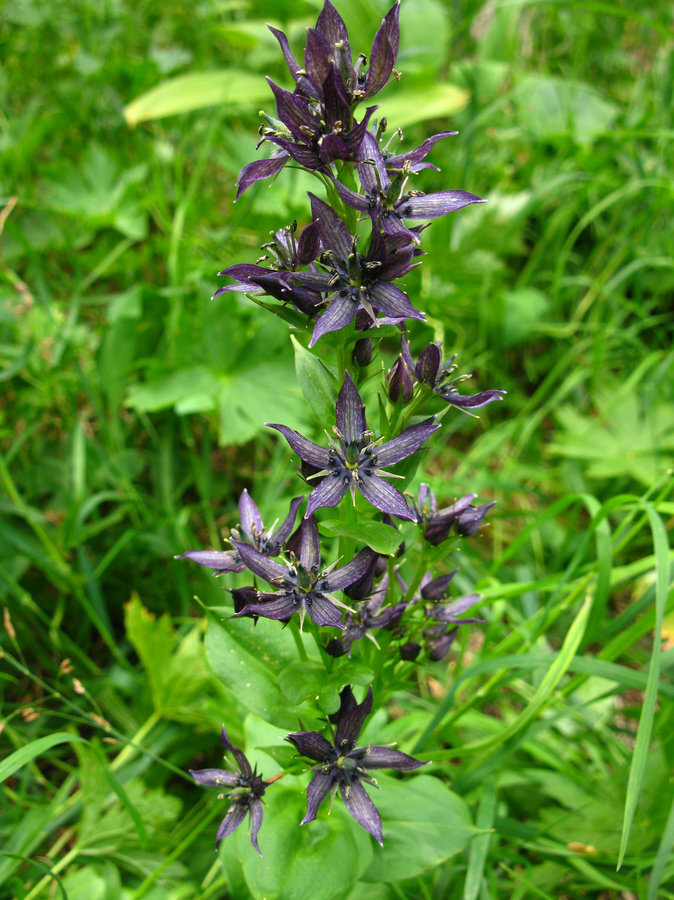
*Swertiabaicalensis*. Photo by the author.

**Figure 2b.**
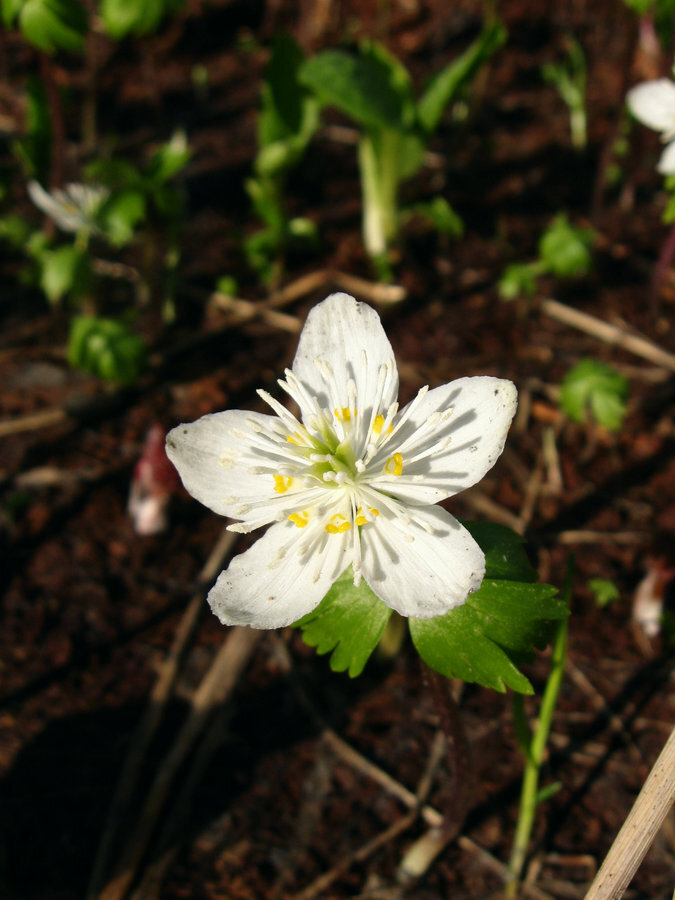
*Eranthistanhoensis*. Photo by the author.

**Table 1.** Top-10 families with the largest number of taxa on species and infraspecific level.

Rank	Family	Number of taxa	% of taxa of the dataset
1	Asteraceae	103	11.77
2	Poaceae	91	10.40
3	Cyperaceae	58	6.63
4	Rosaceae	50	5.71
5	Fabaceae	44	5.03
6	Ranunculaceae	39	4.46
7	Caryophyllaceae	36	4.11
8	Brassicaceae	34	3.89
9	Ericaceae	24	2.74
10	Apiaceae	23	2.63

**Table 2.** Top-10 families with the largest number of occurrences

Rank	Family	Number of occurrences	% of records of the dataset
1	Pinaceae	3534	9.01
2	Poaceae	3331	8.49
3	Asteraceae	3275	8.35
4	Rosaceae	3149	8.03
5	Ericaceae	2677	6.82
6	Ranunculaceae	2300	5.86
7	Betulaceae	1579	4.02
8	Cyperaceae	1252	3.19
9	Fabaceae	1154	2.94
10	Apiaceae	1021	2.60

## References

[B7495141] Abramova L. A., Volkova P. A., Pykhalova T. D., Anenkhonov O. A. (2009). Additions to the cadastre of flora of Baikalsky State Reserve. Turczaninowia.

[B7494541] Abramova L. A., Volkova P. A. (2011). Сосудистые растения Байкальского заповедника (Аннотированный список видов).

[B7495236] Aksenov D. E., Dobrynin D. V., Dubinin M. Ju., Yegorov A. V., Isaev A. S., Karpachevsky M. L., Lestadius L. G., Potapov P. V., Purekhovsky A. Zh., Turubanov S. A., Yaroshenko A. Ju. (2003). Атлас малонарушенных лесных территорий России.

[B7495814] Anenkhonov O. A., Pykhalova T. D., Osipov K. I., Sekulich I. R., Badmayeva N. K., Namzalov B. B., Krivobokov L. V., Munkueva M. S., Sutkin A. V., Tubshinova D. B., D.Ya. Tubanova (2001). Определитель растений Бурятии.

[B7574746] Brianskaia E., Sandanov D., Li Y., Wang Z. (2021). Distribution of alpine endemic plants of northern Asia: a dataset. Biodiversity Data Journal.

[B7620729] Chepinoga V. V., Stepantsova N. V., Grebenjuk A. V., Verkhozina A. V. (2008). Конспект флоры Иркутской области (сосудистые растения).

[B7574782] Chepinoga V. V., Petukhin V. A., Stalmakova D. P. (2017). Grid maps of the compendium "Flora of Central Siberia" (1979) in digital format: outcomes and prospects of application. Растительный мир Азиатской России.

[B7629937] Chepinoga V. V., Protopopova M. V., Pavlichenko V. V. (2017). Detection of the most probable pleistocene micro­refugia on the northern macroslope of the Khamar-Da­ban Ridge (Southern Prebaikalia). Contemporary Problems of Ecology.

[B7495112] Chepinoga V. V., Lashchinskiy N. N., Arbusova G. A., Gladkikh E. M. (2019). New and rare plant species on the Khamar-Daban Range (south of Eastern Siberia). Turczaninowia.

[B7495019] Epova N. A. (1956). Relics of tertiary broad-leaved forests in the Siberian fir (*Abiessibirica*) taiga woods of Khamar-Daban.. Известия Биолого-Географического Научно-Исследовательского Института при Иркутском Государственном Университетете Имени А.А. Жданова [News of Research Institute of Geography and Biology of A.A. Zhdanov Irkutsk State University].

[B7495028] Epova N. A. (1957). Materials on the characterization of the alpine meadows of Khamar-Daban (preliminary report). Известия Биолого-Географического Научно-Исследовательского Института при Иркутском Государственном Университетете Имени А.А. Жданова [News of Research Institute of Geography and Biology of A.A. Zhdanov Irkutsk State University].

[B7495054] Epova N. A., Baranov P. A. (1960). Problems of botany. Materials for the study of flora and vegetation of highlands..

[B7495067] Epova N. A. (1961). To the characterization of the Siberian fir (*Abiessibirica*) taiga forests of Khamar-Daban. Труды Бурятского Комплексного Научно-Исследовательского Института Сибирского отделения Академии наук СССР, Серия Биолого-Почвенная [Proceedings of Buryat Integrated Research Institute of Siberian Branch of Academy of Sciences of USSR. Biology and Soil Series].

[B7495752] Erst A. S., Sukhorukov A. P., Mitrenina E. Yu., Skaptsov M. V., Kostikova V. A., Chernisheva O. A., Troshkina V. I., Kushunina M., Krivenko D. A., Ikeda H., Xiang K., W. Wang. (2020). An integrative taxonomic approach reveals a new species of *Eranthis* (Ranunculaceae) in North Asia. PhytoKeys.

[B7494559] Gamova N. S., Dudov S. V. (2012). *Carexlaevissima* Nakai – new species for the Siberian flora and other new data about flora of the Baikalsky reserve. Turczaninowia.

[B7494568] Gamova N. S., Krasnopevtseva A. S. (2013). Floristic findings in the Baikalsky Reserve. Turczaninowia.

[B7494713] Gamova N. S., Dudov S. V. (2018). Floristic findings in Baikalsky reserve and its protective zone. Proceedings of the Mordovia State Nature Reserve.

[B7494663] Gamova N. S., Dudov S. V. (2018). Additions to the flora of Baikal Nature Reserve. Turczaninowia.

[B7494704] Gamova N. S., Chepinoga V. V., Dudov S. V., Serebryanyi M. M. (2018). Floristic records in Southern part of Baikal region. Bulletin of Moscow Society of Naturalists. Biological series.

[B7494672] Gamova N. S., Dudov S. V., Sutkin A. V., Krasnopevtseva A. S. (2018). New and rarely found in Buryatia taxa of adventive plants from the buffer zone of the Baikal Nature Reserve. Turczaninowia.

[B7494722] Gamova N. S., Kazanovsky S. G., Anenkhonov O. A., Tupitsyna N. N., Olonova M. V., Yurtzeva O. V., Gamova N. S., Shekhovtsov A. I., Kitaev A. V. (2019). *Cotoneasterlucidus* Schltdl., *Epipactishelleborine* L. and another new records from the Baikalsky reserve. Role of scientific research in management and developement of special protected natural areas. Proceedings of scientific conference dedicated to 50th anniversary of Baikalsky Reserve.

[B7495727] Gamova N. S. (2021). Baikalsky State Nature Biosphere Reserve and its buffer zone: floristic data. https://www.gbif.org/dataset/11512fe2-50cf-48e1-b681-3bb1f347eb86.

[B7616976] Gerasimov I. P. (1965). Предбайкалье и Забайкалье.

[B7495076] Ivanova M. M. (1967). Composition, features and some aspects of the genesis of the high-mountain flora of Khamar-Daban (southern Baikal region). Научные Чтения Памяти М.Г. Попова [Scientific Lectures in Memory of M.G. Popov].

[B7494681] Ivanova M. M. (1991). New findings in the flora of the Southern Transbaikalian region and of the Baikal region. Botanicheskii Zhurnal.

[B7494623] Ivanova M. M., Kazanovsky S. G., Kiseleva A. A. (2016). New findings in the flora of the south-eastern shore of the lake Baikal (region of Khamar-Daban mountain range): the nemoral relicts of tertiary flora and rare species. Turczaninowia.

[B7616968] Kartushin V. M. (1969). Агроклиматические ресурсы юга Восточной Сибири.

[B7495132] Krasnopevtseva A. S., Krasnopevtseva V. M., Martusova E. G. (2008). The news of vascular flora of the Baikalsky Reserve. Turczaninowia.

[B7494551] Krasnopevtseva A. S, Martusova E. G., Krasnopevtseva V. M. (2008). Кадастр сосудистых растений Байкальского государственного биосферного природного заповедника.

[B7494690] Krasnopevtseva A. S., Krasnopevtseva V. M., Ananin A. A. (2012). New species in the flora of vascular plants of Baikal Reserve. History and perspectives of the Russian reserve management: problems of protection, scientific research and environmental education: Materials of the scientific-practical conference with international participation, dedicated to the 95th anniversary of the organization of the Barguzin State Natural Biosphere Reserve and the Year of Russian History.

[B7630262] Ladeishchikov N. P., Filippov A. H., Zedgenidze E. G., Zusman I. K., Obolkin V. A., Reznikova S., Ladeishchikov N. P. (1977). Структура и ресурсы климата Байкала и сопредельных пространств (Structure and resources of climate of the Lake Baikal and adjacent territories).

[B7630363] Ogureeva G. N., Miklyaeva I. M., Safronova I. N., Yurkovskaya T. K. (1999). Зоны и типы поясности растительности России и сопредельных территорий. Пояснительный текст и легенда к карте..

[B7618059] Popov M. G., Busik V. V. (1966). Конспект флоры побережий озера Байкал.

[B7616960] Potapov, Zhuravleva, Yaroshenko Intact Forest Landscapes. http://intactforests.org/world.webmap.html.

[B7495781] Seregin A. (2021). Moscow University Herbarium (MW).

[B7494654] Sutkin A. V., Martusova E. G., Krasnopevtseva A. S., Krasnopevtseva V. M. (2016). New data on alien vascular plants in Republic of Buryatia. Turczaninowia.

[B7495103] Sutkin A. V., Krasnopevtseva A. S. (2020). New records of adventive vascular plants in Republic of Buryatia. Turczaninowia.

[B7630371] Takhtajan A. L. (1986). Floristic Regions of the World.

[B7616984] Tjulina L. N. (1976). Влажный Прибайкальский тип поясности растительности.

[B7495094] Tupitsyna N. N., Chepinoga V. V. (2016). Inventory of Hawkweeds (*Hieracium* and *Pilosella*, Asteraceae) in Baikal Siberia. Turczaninowia.

[B7634778] Center Unesco World Heritage Unesco World Heritage Center. http://whc.unesco.org/.

[B7494632] Vasilchenko Z. A., Ivanova M. M., Kiseleva A. A., Malyschev L. I., Peschkova G. A. (1978). Flora of Cisbaikalia.

[B7494645] Verkhozina A. V., Kazanovsky S. G., Stepantsova N. V., Krivenko D. E. (2013). Floristic findings in the republic of Buryatia and Irkutsk region. Turczaninowia.

[B7495085] Volotovsky K. A., Ermolenko E. D. (1985). Addition to the flora of vascular plants of Baikalsky State Reserve. Herald of Kharkiv University.

